# Entomopathogens and Parasitoids Allied in Biocontrol: A Systematic Review

**DOI:** 10.3390/pathogens12070957

**Published:** 2023-07-20

**Authors:** Janique Koller, Louis Sutter, Jérémy Gonthier, Jana Collatz, Lindsey Norgrove

**Affiliations:** 1 Agroscope, Plant-Production Systems, Route des Eterpys 18, 1964 Conthey, Switzerland; janiquestephanie.studer@students.bfh.ch (J.K.); louis.sutter@agroscope.admin.ch (L.S.); 2 School of Agricultural, Forest and Food Sciences (HAFL), Bern University of Applied Sciences (BFH), Länggasse 85, 3052 Zollikofen, Switzerland; 3 Agroscope, Agroecology and Environment, Reckenholzstrasse 191, 8046 Zurich, Switzerland; jeremy.gonthier@agroscope.admin.ch (J.G.); jana.collatz@agroscope.admin.ch (J.C.)

**Keywords:** pest management, microbial pesticide, parasitoid wasp, compatibility, synergism, antagonism

## Abstract

Biological pest control is an environmentally friendly alternative to synthetic pesticides, using organisms such as viruses, bacteria, fungi, and parasitoids. However, efficacy is variable and combining different biocontrol agents could improve success rates. We conducted a systematic review of studies combining a parasitoid with an entomopathogenic microorganism, the first of its kind. We searched in Web of Science and extracted data from 49 publications matching the pre-defined inclusion criteria. Combinations of 36 hymenopteran parasitoids with 17 entomopathogenic microorganisms used to control 31 target pests were found. *Trichogramma pretiosum* and *Encarsia formosa* were the most frequently studied parasitoids, while *Beauveria bassiana*, *Metarhizium anisopliae*, *Lecanicillium muscarium*, *Bacillus thuringiensis* var. *kurstaki*, the Spodoptera exigua multiple nucleopolyhedrovirus, and the Spodoptera frugiperda multiple nucleopolyhedrovirus were the main microbial agents assessed. Out of 49 parasitoid–microorganism combinations assessed in the laboratory experiments, thirty-eight were reported as compatible and six as incompatible. Timing and dosage of biopesticides played a crucial role, with later application and appropriate dosage minimizing adverse effects on parasitoid development. More research is needed to assess compatibility and efficacy under real-world conditions. Our review provides valuable insights for researchers and practitioners to optimize the combined use of micro- and macroorganisms for effective pest control.

## 1. Introduction

Biological pest control is an alternative to synthetic pesticides with fewer adverse environmental effects [1]. Among the most used organisms in biological control are microorganisms such as entomopathogenic bacteria, fungi, and viruses. *Bacillus thuringiensis* sp. (Bt) are the most widely used bacteria to control pests in agriculture, forestry, and public health [2]. They release toxins that cause cell lysis and death after binding with specific receptors in the insect midgut [3]. Entomopathogenic bacteria have a wide host range, mainly lepidopteran, dipteran, and coleopteran, but were reported to have minimal to no negative effects on beneficial organisms [3]. *Beauveria bassiana* (Balsamo) Vuillemin, *Metarhizium* sp., *Paecilomyces farinosus* (Holm ex S.F. Gray) Brown & Smith, and *Lecanicillium muscarium* Zare & Gams (previously known as *Verticillium lecanii* (Zimmermann) Viegas) are the most used fungi for insect biocontrol. They attack pests by damaging their integument or gut epithelium, using nutrients in their hemocoel, or releasing toxins [2,3]. They are ubiquitous in the environment, have a broad range of arthropod hosts, and often cause epizootics in insect populations. Compatibility with arthropod predators and parasitoids should be tested to ensure compatibility and maximize efficacy [3]. Approximately a dozen viral bioinsecticides are commercially available, and currently only nucleopolyhedroviruses and granuloviruses specifically target lepidopteran pests. Viruses enter the host cells, replicating in the nuclei or cytoplasm before causing cell lysis and sometimes enzootics [2,3]. Another key group for all types of biocontrol are parasitoids. Most of them belong to the orders of the Hymenoptera and Diptera, fewer to the orders of the Coleoptera, Lepidoptera and, Neuroptera [4]. Their larvae develop on or in the body of other arthropods and usually kill them by their feeding. Some parasitoids parasitize eggs, while others parasitize larvae, pupae, or even adults. *Trichogramma* sp. are the commercially most used parasitoids, which develop in the eggs of Lepidoptera [5,6]. *Encarsia formosa* is commonly used against whiteflies and *Aphidius colemani* against aphids [7].

**Figure 1:**
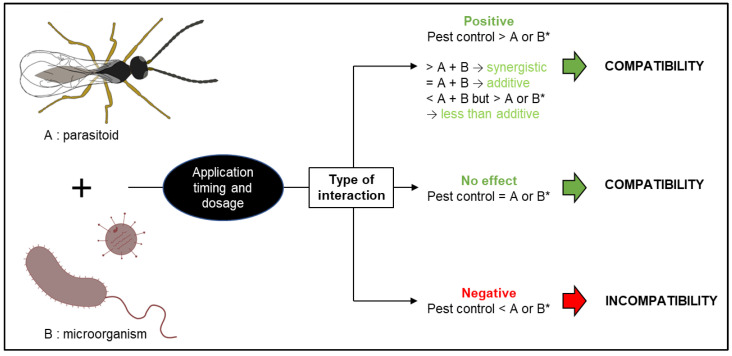
Types of interactions and factors influencing the compatibility of entomopathogenic microorganisms and parasitoids. * Comparison made with the more effective of agents **A** or **B**.

The success of biological control relies on multiple biotic and abiotic factors. For example, entomopathogenic microorganisms are susceptible to climatic conditions such as temperature, humidity, and UV radiation [8]. Likewise, parasitoid longevity and efficacy rely on factors such as host density, nectar and pollen sources, habitat composition, and climatic conditions [9]. With some of these factors being difficult to manage, biological control can be of variable efficacy and reliability. Combining different biocontrol agents could minimize that problem (Figure 1). Roy and Pell [10] conducted a narrative review on interactions between entomopathogenic fungi (EF) and other natural enemies. They found that predators and parasitoids may foster the development of epizootics by vectoring EF and causing increased movement of infected hosts. Several key factors that influence potential antagonistic effects when combining parasitoids with EF were mentioned: (i) fungal dosage, (ii) relative timing of parasitism and fungal infection, and (iii) fungal identity. More recently, Quesada-Moraga et al. [11] reviewed the compatibility between EF and parasitoids with mixed results. Some studies concluded that parasitoids serve as vectors of EF, even showing synergistic interactions. Other studies found that previous inoculation with EF can impact the fitness of parasitoids, shortening their lifetime yet increasing oviposition rates. Most studies concluded that the combination is beneficial when, as underlined by Roy and Pell [10], release times are adjusted appropriately, with the timing and order of agent administration being crucial. Cossentine [12] reviewed the interactions between baculoviruses and parasitoids. In laboratory experiments, parasitoids could reduce the pathogenicity of baculoviruses in hosts. Yet, in field trials, parasitoids did not reduce the overall mortality caused by an applied baculovirus. Indeed, parasitoids can spread or accelerate the spread of the virus within hosts, increasing efficacy under field conditions. Baculoviral infections can lower parasitoid population densities, but many parasitoids can avoid or reduce their use of virus-infected hosts, and a strategically timed baculoviral biopesticide should have a low impact on host–parasitoid populations. The impact of Bt-bioinsecticides on parasitoids has been reviewed recently [13] with the conclusion that combining parasitoids with Bt-bioinsecticides could significantly increase crop yield and improve pest control. However, the impact of Bt on beneficial arthropods is still being studied due to the high number of Cry toxins untested against them. It is particularly important to consider indirect impacts of these products on parasitoid physiology and behaviour [13].

Given the increasing interest in biological pest control in the past decade and the growing importance of entomopathogen agents [14,15], a new and systematic review of the literature combining all different entomopathogens is timely. We aimed to synthesize the state-of-the-art when combining a parasitoid with an entomopathogenic microorganism. Combining parasitoids and microorganisms may be positive, negative, or with no effect on pest control compared to their use alone. We hypothesized that the effects could be positive and that more efficient pest control could be achieved through combining agents. We, therefore, assessed: (1) Which are the most studied combinations of parasitoids and microorganisms? (2) Which combinations of microorganisms and parasitoids are compatible? (3) How do microorganisms influence the life table parameters of parasitoids? (4) Which key factors influence the compatibility of microorganisms and parasitoids? (5) Can more efficient pest control be achieved by combining a parasitoid with a microorganism instead of using them individually?

## 2. Materials and Methods

### 2.1. Search Criteria

We used the following search string in Web of Science Core Collection on 2 March 2023: ((fung* OR vir* OR entomopathog* OR “vir*-based insecticid*” OR “fung*-based insecticid*” OR “biological insecticid*” OR “microbial insecticid*” OR “natural insecticid*”) AND (biocontrol OR biological control)) AND (*parasit* AND (biocontrol OR biological control)) AND (combin* OR interaction OR substitut* OR synergist* OR antagonist*) AND (“integrated pest control” OR “biological pest control” OR “pest biocontrol” OR “pest populations” OR pest OR “pest management” OR IPM). We did not use any date limitation but confined our search to journal articles in English.

### 2.2. Data Inclusion and Exclusion Criteria

We assessed articles by analysing abstracts following the Preferred Reporting Items for Systematic Reviews and Meta-Analyses (PRISMA, [16]) (Figure S1). We obtained 547 initial hits, of which 121 were excluded as they were review articles. Based on other pre-defined exclusion criteria, 377 further articles were excluded. Inclusion of a publication was based on three criteria: (1) the study was an experiment concerning the biological control of a pest; (2) the experiment combined at least a parasitoid and an entomopathogenic fungus, bacterium, or virus; and (3) the study design included at least a no-treatment control. Studies combining entomopathogenic nematodes with parasitoids were excluded. Laboratory, semi-field, field, and greenhouse experiments were considered, but modelling and simulations were excluded.

### 2.3. Data Extraction

We used Citavi software (version 6.11.0.0) to import the included studies. We extracted data on (1) pest, (2) control agents, (3) crop, (4) location, (5) study design, (6) evolution of pest and biocontrol agent populations, (7) crop damage, (8) crop yield, and (9) compatibility of biocontrol agents from the 49 selected articles [17,18,19,20,21,22,23,24,25,26,27,28,29,30,31,32,33,34,35,36,37,38,39,40,41,42,43,44,45,46,47,48,49,50,51,52,53,54,55,56,57,58,59,60,61,62,63,64,65]. Each combination of biocontrol agents was considered an experiment, leading to 100 distinct experiments, as some publications studied multiple combinations simultaneously. Different strains of the same microorganism combined with one parasitoid were also counted as distinct experiments. Data from experiments assessing multiple dosages, timing of application, and types of exposition of the parasitoid to the microbial biocontrol agent were extracted as several observations. Each different treatment was considered as a single observation leading to the extraction of 484 individual observations.

### 2.4. Data Synthesis and Analysis

Most studies reported the effect of entomopathogenic microorganisms on the life table parameters of parasitoids. In these studies, parasitoids were the dependent variable. The impact of a treatment with entomopathogenic microorganisms was usually compared with a “no-treatment control” (parasitoids only). Data were synthesized by “vote counting” of the numbers of positive, neutral, and negative effects caused by the entomopathogen on each studied parameter of the parasitoid’s fitness. We used “positive” when the microorganism treatment significantly improved the development of the parasitoid in comparison to the no-treatment control. “Negative” was used when the microorganism treatment significantly hindered the development of the parasitoid compared to the no-treatment control. “No effect” was used when no significant difference was found between the treatment and the parasitoid-only control. In a simplified way, combinations with positive or no effect were defined as compatible. However, in the reviewed papers, compatibility was assessed by an overall analysis of all the studied parameters. Being unable to synthesize all interconnected parameters for all reviewed papers, we reported compatibility based on the authors’ conclusions. For example, if they mentioned that two biocontrol agents could be combined at a defined timing and dosage despite observed antagonistic effects under other conditions, we reported those as compatible.

When the effect on pest control was reported, “positive” was used to categorize when the pest reduction achieved by the combined biocontrol agents was higher than the reduction achieved by the strongest agent applied individually. Positive effect can be either “synergistic” when the pest reduction achieved by the combined biocontrol agents is higher than the addition of that achieved by each agent applied individually, “additive” when the pest reduction achieved by the combination is equal to the addition of that achieved by each agent used alone, or “less than additive” when the pest reduction achieved by the combined biocontrol agents is significantly higher than that achieved by each agent used alone, yet lower than additive. “No effect” was used when no significant difference was observed between the pest reduction achieved by the combined agents and that achieved by the strongest agent applied individually. “Negative” was used when the combined biocontrol agents achieved a lower pest reduction than the strongest agent used individually.

Among the publications selected for this review, reports on laboratory combination experiments were predominant. From those publications, we extracted life-history data on sixteen parameters for parasitoids, four parameters for pests, and five parameters for entomopathogenic microorganisms. For further analysis, we focused on the parasitism rate, emergence rate, mortality, sex ratio, and longevity of parasitoids, as these were the most documented parameters.

The packages ggplot2 [66], tidyverse [67], and webr [68] in RStudio (version 4.1.2), as well as Microsoft Excel (version 2208), were used to obtain descriptive statistics and to visualize data.

## 3. Results and Discussion

### 3.1. Scope of the Publications

Forty-nine studies detailing one hundred combination experiments were conducted from 2000 to 2022. Eighty-four were laboratory experiments and thus formed the focus of our results. In addition, nine were field experiments, five were semi-field experiments, and two were greenhouse experiments. All combination experiments included a no-treatment control as it was an inclusion criterion. Four experiments included additional controls with either the parasitoid (3) or microorganism (1) alone, and fourteen included both types of controls. Six experiments included a synthetic insecticide control in addition to the no-treatment control.

**Figure 2:**
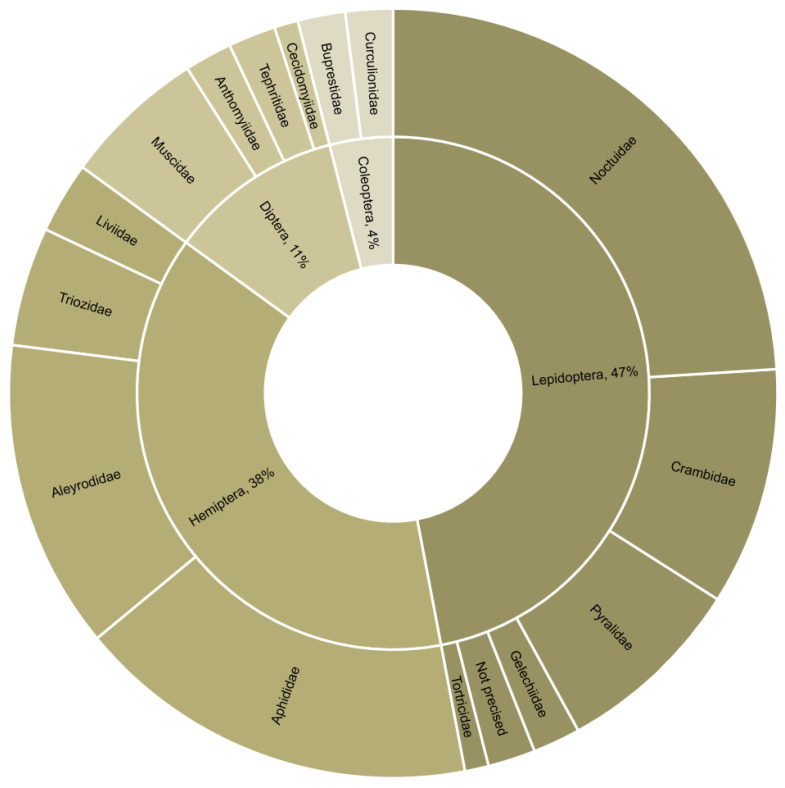
Order and family of target pests where combined biocontrol agents were used (*n* = 100 combination experiments from 49 studies).

**Figure 3:**
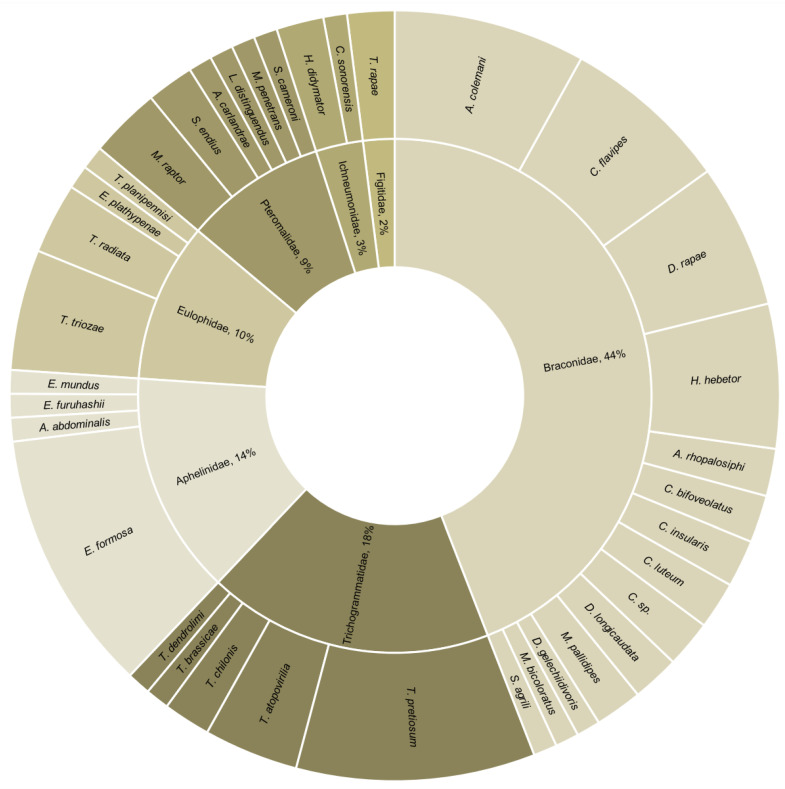
Family and species of hymenopteran parasitoids used in combination with an entomopathogenic microorganism (*n* = 100 combination experiments from 49 studies).

**Figure 4:**
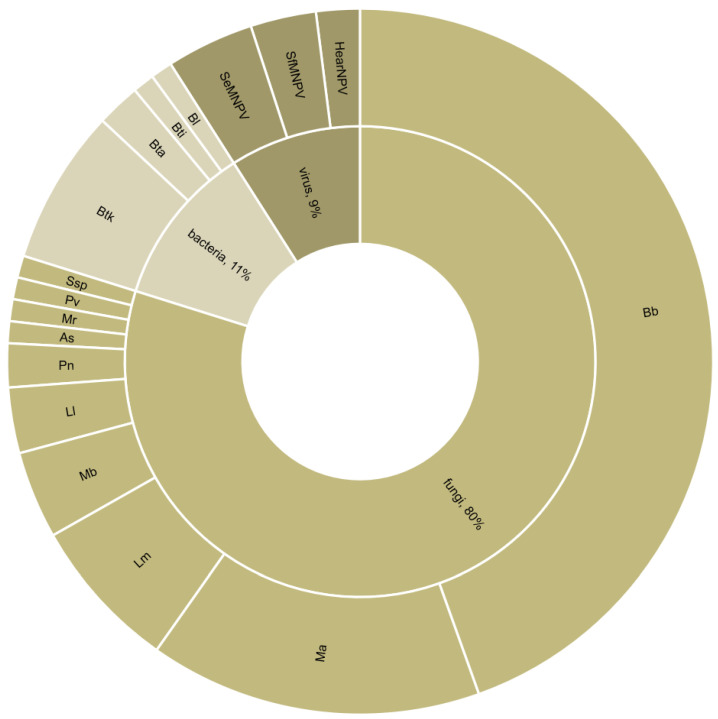
Type and species of entomopathogenic microorganisms used in combination with a parasitoid (*n* = 100 combination experiments from 49 studies). Bb = *Beauveria bassiana*; Ma = *Metarhizium anisopliae*; Lm = *Lecanicillium muscarium*; Mb = *Metarhizium brunneum*; Ll = *Lecanicillium longisporum*; Pn = *Pandora neoaphidis*; As = *Acremonium sclerotigenum*; Mr = *Metarhizium robertsii*; Pv = *Paecilomyces variotii*; Ssp. = *Simplicillium* sp.; Btk = *Bacillus thuringiensis* var. *kurstaki*; Bta = *Bacillus thuringiensis* var. *aizawai*; Bti = Bt var. *israelensis*; Bl = *Brevibacillus laterosporus*; SeMNPV = Spodoptera exigua multiple nucleopolyhedrovirus; SfMNPV = Spodoptera frugiperda multiple nucleopolyhedrovirus; HearNPV = Helicoverpa armigera nuclopolyhedrovirus.

The reviewed studies dealt with 31 target pests. Approximately half of these were Lepidoptera, of which 52% were Noctuidae. The remaining were Hemiptera, Diptera, and Coleoptera (Figure 2). The studied biocontrol agents included 36 parasitoids and 17 entomopathogenic microorganisms. All parasitoids were Hymenoptera, and the most represented families were Braconidae (44%) and Trichogrammatidae (18%) (Figure 3). Most combination experiments were conducted with fungi (80%), followed by bacteria (11%) and viruses (9%) (Figure 4).

### 3.2. Assessed Combinations of Biocontrol Agents in Laboratory Experiments

The conducted laboratory experiments reported entomopathogenic microorganisms’ effects on parasitoid’s life table parameters. In total, 49 combinations were tested in 84 laboratory experiments. While many experiments were conducted with fungi, few analysed the compatibility of viral and bacterial biocontrol agents with parasitoids (Table 1). *B. bassiana* was part of all the most frequent combinations with *Trichogramma pretiosum* [24,52], *Tamarixia triozae* [63,64], *E. formosa* [40,51], and *Trichogramma atopovirilia* [24]. *Metarhizium anisopliae* (Metschnikoff) used with *Cotesia flavipes* [55,58] was the next most assessed combination. All other combinations appeared in one to three laboratory experiments each. *Trichogramma pretiosum* [24,47,52] and *E. formosa* [29,38,40,51] were the most researched parasitoids, followed by *A. colemani* [23,30,32,35,46], *C. flavipes* [55,58], and *Diaeretiella rapae* [18,43]. In terms of microorganisms, *B. bassiana* [17,18,24,26,32,33,35,40,42,43,48,51,52,54,55,57,58,59,60,61,63,64,65] was the most studied fungus, followed by *M. anisopliae* [30,34,36,37,48,49,50,51,53,55,57,58] and *L. muscarium* [18,23,38,41,46]. *Bacillus thuringiensis* var. *kurstaki* (Btk) [19,21,22,47,56] was the most frequently assessed bacterium. Among viruses, the *Spodoptera exigua* multiple nucleopolyhedrovirus (SeMNPV) [25,39,62] and the *Spodoptera frugiperda* multiple nucleopolyhedrovirus (SfMNPV) [27,28] were the most frequently tested in combination with parasitoids.

### 3.3. Reported Compatibility of Biocontrol Agents Assessed in Laboratory Experiments

Thirty-eight out of forty-nine combinations of biocontrol agents were reported as compatible [18,19,20,22,24,25,26,29,32,34,35,36,37,39,41,43,44,45,47,49,50,52,53,54,55,56,57,58,59,60,62,63,64,65] (Table 1). Six combinations were reported as incompatible [17,27,28,42,51], often due to a lower emergence rate caused either by bad timing, direct infection, or too high dosage. No answer about compatibility was given for the five remaining ones [30,38,54,56]. Divergent results reported from the combination of *L. muscarium* with *A. colemani*. Aqueel and Leather [23] found that these biocontrol agents interacted negatively. In contrast, Mohammed and Hatcher [46] reported them as compatible as long as the fungus was applied more than five days after parasitoid release.

The influences of combined biocontrol agents on pest mortality were analysed in ten laboratory experiments extracted from nine studies [25,28,38,44,45,50,57,62,65]. Out of 41 observations made in these experiments, 14 reported a positive effect, with significantly higher pest mortality when biocontrol agents were combined compared to the strongest agent used alone. In 11 of these, the interaction was less than additive [25,28,57,65]. In two further observations, it was additive [50], and in one, it was synergistic [50]. Twenty-six further observations reported that the combination had no effect on pest mortality [25,38,44,45,62], and one single study reported a negative effect [25].

Out of 266 observations, 83 (31%) reported a negative effect of entomopathogenic microorganisms on parasitism rate [17,24,37,38,39,42,43,44,45,46,51,52,55,60,65] (Table 2). This parameter was positively influenced in four observations (2%) [20,24,52,54]. The other studies observed no significant differences between the treatment and control [19,20,22,23,24,28,32,35,36,39,43,44,45,46,47,52,53,54,55,58,60,63,65]. The parasitism rate was often related to the ability of the parasitoid to discriminate against infected hosts. Females appeared to avoid ovipositing on treated hosts mainly when they had a choice between treated and healthy hosts. Under no-choice conditions, females only laid fewer eggs on treated hosts than on healthy ones in one out of seventy observations (~1%) [19,20,38,57,63]. In contrast, when females had a choice, they avoided treated hosts for oviposition in 15 out of 35 observations (43%) [19,20,32,37,39,44,45,46,47,54,65]. It is important to stress that sixty-three out of the seventy observations made under no-choice conditions were extracted from a single study reporting three experiments [63]. Therefore, further research needs to be done to confirm the above statements. Discrimination of infected hosts would be beneficial under field conditions. Indeed, parasitoids could complement the effects of entomopathogenic microorganisms on the pest while avoiding the negative effects of the latter on themselves.

A reduced emergence rate of parasitoids combined with a microbial biocontrol agent was reported in 144 of 257 observations (56%) [17,18,19,20,23,24,25,26,27,28,29,30,34,36,37,38,39,42,43,44,45,46,49,50,51,52,53,54,55,56,57,58,59,60,62,63,64,65]. Entomopathogenic microorganisms had no significant influence on this parameter in all other observations [19,20,22,24,28,29,32,35,39,41,43,46,47,52,53,55,56,57,60,65]. Parasitoids combined with microbial agents had higher mortality in seventy-one out of one hundred ninety-four observations (37%) [18,24,26,27,28,29,39,49,54,55,56,57,59,65] and lower mortality in four further observations (2%) [55]. No significant effect was reported in the remaining ones [18,24,26,28,39,41,47,49,53,54,55,56,57,63,65]. The contact with entomopathogenic microorganisms reduced the female offspring sex ratio of parasitoids in 17 out of 92 observations (18%) [23,24,46,52]. No significant change of this parameter was observed in all other cases [17,24,28,32,35,41,43,46,52,53,55,57,65]. Female parasitoids combined with microbial biocontrol agents had shorter longevity in 67 out of 130 observations (52%) [17,19,20,24,42,43,52,55,56,57,64]. A single observation (1%) reported higher longevity of female *Trichogramma chilonis* when fed with a mixture of honey and Btk in comparison with females fed pure honey [22]. No significant difference in female longevity was reported in all other observations [19,20,22,24,29,34,35,42,46,50,52,53,55,56]. When combined with microbial biocontrol agents, male parasitoids had shorter longevity in 55 out of 125 observations (44%) [17,19,24,42,52,55,64] (Table 2). This parameter remained unchanged in all other observations [17,19,20,24,29,34,35,41,42,50,52,53,55,56].

Timing of application and dosage of biopesticides were important factors influencing the compatibility of entomopathogenic microorganisms with parasitoids. In total, the importance of the timing of application was emphasized in 44 out of the 84 laboratory experiments (52%) [17,20,21,24,25,27,28,29,34,36,38,39,41,43,45,46,50,51,52,57,60,62,63,64,65] (Table 1). The importance of dosage was highlighted in 25 out of these 84 experiments (30%) [19,20,22,24,25,29,39,41,49,54,60,62,64,65]. For example, *B. bassiana* was reported as compatible with parasitoids in 34 out of 37 experiments [17,18,24,26,32,34,35,42,43,51,52,54,55,58,59,60,63,64,65], but the importance of 1) an adapted application timing and 2) dosage was mentioned in 25 (68%) [17,24,34,43,51,52,60,63,64,65] and 13 (35%) [24,54,60,64,65] of these, respectively. The optimal dosage differed according to the target pest and the combination of biocontrol agents used. It must be sufficient to kill the pest without negatively affecting the parasitoid. The interval length between parasitoid release and infection also differed and needed to be defined for each pair of biocontrol agents.

In most reviewed publications, applying the entomopathogenic microorganism after parasitism was recommended to reduce its negative effects on parasitoid development. Waiting for 24 h after the emergence of *T. pretiosum* before applying *B. bassiana* reduced the negative effects of the entomopathogenic fungus on the parasitoid [52]. *Beauveria bassiana* and *T. trizoae* were assessed as compatible if applied at different times [63]. Infection rate of this parasitoid by the fungus was significantly higher in early instars than in more advanced developmental stages [64]. Therefore, the parasitoid should be released before applying the fungus [17,64]. The same conclusion was obtained for *B. bassiana* and *M. anisopliae* used in combination with *T. trizoae* [34]. When combined with *Aphelinus abdominalis*, *B. bassiana* should be applied only when most parasitoids already transformed into pupae and are less susceptible to the fungus [60].

Similarly, the first application of *Lecanicillium longisporum* (Petch) Zare & Gams should be conducted one day after *E. formosa* enters the pupal stage to reduce competition between the biocontrol agents [29]. Detrimental effects of *B. bassiana* and *M. anisopliae* on the development of *E. formosa* could be reduced by waiting at least four days after parasitoid release to spray the microbial agents [51]. Post-parasitism application of *Metarhizium brunneum* Petch also appeared to be best suited for *Hyposoter didymator* as it limited negative effects on the parasitoid due to direct contact with the fungus [45]. A spatial separation of the microbial treatment and the parasitoid release is also possible to avoid these kinds of effects [53]. Fewer *A. colemani* with a lower rate of females emerged from aphids treated with *L. muscarium* within five days of parasitization. In contrast, fungal application six or seven days after aphids had been parasitized did not significantly affect the development, emergence rate, or sex ratio of the parasitoid [46]. Four different time intervals between parasitism by *Campoletis sonorensis* and application of the SfMNPV were tested. Decreasing parasitoid mortality was observed with increasing time interval. The virus did not affect the survival of *C. sonorensis* when applied six days after parasitization [28]. In an experiment combining *Euplectrus plathypenae* and the SeMNPV, the parasitoid was only able to complete its development when the viral infection occurred at least two days after parasitization [46].

In contrast, few recommendations to apply the entomopathogenic microorganism prior to the parasitoid release were found in the reviewed publications. *Bacillus thuringiensis* var. *kurstaki* and the *Helicoverpa armigera* nucleopolyhedrovirus (HearNPV) were recommended to be applied two days before releasing *H. hebetor* to control *Helicoverpa armigera* on chickpeas [20]. Similarly, it was recommended to apply *B. bassiana* before releasing *Trichogramma dendrolimi* so that pest eggs unaffected by the fungal treatment become parasitized [65].

Here, we show for the first time that most studied combinations of biocontrol agents are compatible under controlled conditions. In the best cases, parasitoids are outside the field of action of entomopathogenic microorganisms and remain unaffected when combined with the latter. If not, the timing of application of the biocontrol agents and the biopesticide dose must be carefully determined. Mathematical models such as the one created by Gonthier et al. [69] for the combined use of *Necremnus tutae* and Phthorimaea operculella granulovirus against *Tuta absoluta* can be helpful tools for this purpose. If the dose required to control a specific pest is higher than that tolerated by the parasitoid, the compatibility of the two biocontrol agents is compromised. In terms of pest control, combined biocontrol agents had a positive influence in most cases compared with each agent used alone. However, in their narrative review, Roy and Pell [10] highlighted the importance of conducting field experiments in addition to laboratory bioassays to assess the physiological and ecological susceptibility of natural enemies in a realistic environment. In the field, unpredictable climatic conditions could modify the dynamics of the biocontrol agents observed in the laboratory. Furthermore, less precise application of biopesticides and broader spatial dispersion of pests and parasitoids could significantly influence the compatibility and efficacy of the combinations of biocontrol agents. The presence of other insect species could also influence the level of pest control achieved by parasitoids and entomopathogenic microorganisms that have a wide host range.

### 3.4. Parasitoid Life History and Susceptibility to Entomopathogens

Parasitoid life history (e.g., generation time, population structure) can influence their susceptibility to entomopathogens [70,71]. As many entomopathogens target the larval stage of the pest, egg parasitoids are less likely to be in contact or compete with entomopathogens, making them de facto more compatible. The development strategies of larval parasitoids can strongly influence their susceptibility to entomopathogens. Endoparasitoids, which lay their eggs inside the host insect’s body, may be less exposed than ectoparasitoids, which lay their eggs on the surface of the host, sometimes in open environments where the microorganisms can directly reach the parasitoid larvae [72]. On the other hand, endoparasitoids typically have a longer development time than ectoparasitoids, which can also influence their susceptibility to entomopathogens. As entomopathogens have a slower mode of action, taking longer to kill the host insect, parasitoids with shorter life cycles may emerge from the host before the entomopathogen has a chance to kill it, reducing their exposure to the pathogen [72].

In fact, endoparasitoids are generally considered to be more susceptible to entomopathogens than ectoparasitoids [73], as the pathogen has more time to act on the immature parasitoid during its extended development period inside the host insect [74]. Additionally, endoparasitoids are more likely to be exposed to entomopathogens that are ingested by the host insect, as the pathogen can spread throughout the host’s body and affect the parasitoid’s physiological function [75].

### 3.5. Investigated Combinations and Reported Compatibility of Biocontrol Agents in Field, Semi-Field and Greenhouse Experiments

Nine field, five semi-field, and two greenhouse experiments reported the effects of fifteen combinations of parasitoids and entomopathogenic microorganisms on pest control. Thirteen experiments included fungi, one included a bacterium, and the two remaining ones were conducted with viruses [21,31,33,39,40,46,48,61]. As in the laboratory experiments, *B. bassiana* was the most studied microorganism. It was tested in combination with *E. formosa* [65], *Chelonus bifoveolatus*, *Coccygidium luteum* and *Cotesia* sp. [48], *Anisoptermalus calandrae* and *Lariophagus distinguendus* [33], and *Macroglenes penetrans* [61] in one experiment, each. In their greenhouse experiment, Labbé et al. [40] found that *B. bassiana* used in addition to *E. formosa* resulted in a higher pest reduction than the parasitoid alone without harming the development of the latter. In contrast, the fungus was reported as incompatible with *A. calandrae* and *L. distinguendus* [33]. Indeed, in semi-field experiments, *B. bassiana* affected both parasitoids negatively, resulting in lower pest control of the fungus–parasitoid combinations compared with the parasitoid released alone. No answer about the compatibility of this fungus with the other parasitoids mentioned above was given.

In the field experiments conducted by Ngangambe and Mwatawala [48], *M. anisopliae* was tested in combination with *C. bifoveolatus*, *C. luteum*, and *Cotesia* sp., also in one experiment each. In this study, biopesticides based on *B. bassiana* and *M. anisopliae* were reported as less harmful to natural parasitoids than synthetic insecticides based on flubendamide. Fuentes-Contreras and Niemeyer [31] assessed *Pandora neoaphidis* and *Aphidius rhopalosiphi* as compatible in two semi-field experiments. Combining these biocontrol agents resulted in more efficient pest control than each agent used alone. It significantly reduced the growth rate of the pest population. In a greenhouse experiment, the SeMNPV and *Microplitis pallidipes* were found to be compatible [39]. The parasitoid vectored the virus, and their combined use resulted in a significantly higher pest control. Thus, it was recommended to expose the parasitoid to the virus before releasing it.

*H. hebetor* was tested in combination with Btk and the HearNPV in one field experiment each [21]. These combinations were reported as compatible. Both parasitoid–microbe combinations significantly reduced pest density and crop damage in comparison with each biocontrol agent alone. Crop yield was significantly increased, but so were the control costs. Yield gain was insufficient to cover the additional treatment costs, meaning that combining the biocontrol agents negatively affected the crop’s profitability. The remaining investigated combinations were *L. muscarium* with *A. colemani* in a semi-field experiment [46] and the SeMNPV with *M. pallidipes* in a greenhouse experiment [39]. Both were reported as compatible and significantly reduced pest density when deployed together.

Here, we show that combining entomopathogenic microorganisms with parasitoids in the field appears beneficial for pest control; however, profitability may be reduced due to increased control costs. However, the interaction of biocontrol agents on key aspects, namely crop damage, crop yield, and treatment costs, were assessed in only two out of the hundred reviewed experiments. These agronomic and financial parameters must be considered in future research assessing the compatibility of parasitoids and entomopathogenic microorganisms.

## 4. Conclusions

Environmental pollution, loss of biodiversity, pest resistances, and risks to human health are among the controversial effects of synthetic pesticides. Alternative methods for pest control are sought after. Combining biocontrol agents can improve pest control and reduce harmful effects on the environment. In this systematic review, we show for the first time that many combinations of parasitoids and entomopathogenic microorganisms are compatible and can be deployed together. Eighty percent of the biocontrol agent combinations included in the reviewed papers were deemed compatible. Combinations, including parasitoids and fungi, were well represented in the literature. In contrast, few experiments were found combining bacteria with parasitoids, despite the large number of Bt-biopesticides. Further research on combining bacterial or viral biopesticides with parasitoids is required. The most studied microorganisms of each category, i.e., *B. bassiana* and *M. anisopliae* for fungi, Btk for bacteria, as well as the SeMNPV and the SfMNPV for viruses, were found compatible with many different parasitoids. However, most of the studies were conducted in the laboratory, and new experiments under field conditions are necessary to include agronomic and financial parameters in the final compatibility assessment. Moreover, most of the reviewed studies focused on the impacts of entomopathogenic microorganisms on parasitoids. Few examined the effects of parasitoids on the development and dissemination of microbial biocontrol agents. Further research is required to analyse how both types of biocontrol agents influence each other in the field. Such an assessment should be conducted on more than one generation of parasitoids to highlight possible long-term effects. Appropriate timing of application and dosage must be defined individually for each combination of biocontrol agents against each specific pest, as these are key success factors. Combining biocontrol agents has the potential for pest control, yet interactions between parasitoids and entomopathogenic microorganisms should be further researched. To develop innovative methods, interdisciplinary work should be fostered. Finally, pest biocontrol methods must be viable. Therefore, the availability and the production costs of biocontrol agents should be assessed and further improved.

## Figures and Tables

**Table 1:** Compatibility of 49 combinations of biocontrol agents extracted from laboratory experiments in the reviewed studies; green = combination reported as compatible; red = combination reported as incompatible; no fill = no report of compatibility; ^t^ = at least one paper mentioning application timing as important; ^d^ = at least one paper mentioning dosage as important; FI. = Figitidae; ICHN. = Ichneumonidae; PTEROM. = Pteromalidae; TRICHOGRAMM. = Trichogrammatidae; NPV = nucleopolyhedrovirus; MNPV = multiple nucleopolyhedrovirus (*n* = 84 experiments from 43 studies [17,18,19,20,22,23,24,25,26,27,28,29,30,32,34,35,36,37,38,39,41,42,43,44,45,46,47,49,50,51,52,53,54,55,56,57,58,59,60,62,63,64,65]).

Combinations of Parasitoids and Entomopathogenic Microorganisms		Aphelinidae				Braconidae											Eulophidae				FI.	ICHN.		PTEROM.			TRICHOGRAMM.					Total
		*A. abdominalis*	*E. formosa*	*E. furuhashii*	*E. mundus*	*A. colemani*		*C. insularis*	*C. flavipes*	*D. longicaudata*	*D. rapae*	*D. gelechiidivoris*	*H. hebetor*	*M. bicoloratus*	*M. pallidipes*	*S. agrili*	*E. plathypenae*	*T. radiata*	*T. triozae*	*T. planipennisi*	*T. rapae*	*C. sonorensis*	*H. didymator*	*M. raptor*	*S. cameroni*	*S. endius*	*T. atopovirilia*	*T. brassicae*	*T. chilonis*	*T. dendrolimi*	*T. pretiosum*	
**Bacteria**	*Bacillus thuringiensis* var. *aizawai*																														2	2
	*Bacillus thuringiensis* var. *israelensis*																							1								1
	*Bacillus thuringiensis* var. *kurstaki*												1 ^d^											1					2 ^d^		2	6
	*Brevibacillus laterosporus*																							1								1
**Fungi**	*Acremonium sclerotigenum*										1																					1
	*Beauveria bassiana*	1 ^td^	5 ^t^		1	2			3		2 ^t^					1		2 ^t^	5 ^td^	1	1 ^d^					2	4 ^td^			1 ^td^	6 ^td^	37
	*Lecanicillium longisporum*		3 ^td^																													3
	*Lecanicillium muscarium*		1^t^	1 ^td^		1 ^t^	2				1																					6
	*Metarhizium anisopliae*		1 ^t^			1			4	1		1 ^t^	2 ^t^					1 ^t^							1 ^d^			1 ^t^				13
	*Metarhizium brunneum*					1															1 ^d^		2									4
	*Metarhizium robertsii*									1																						1
	*Paecilomyces variotii*										1																					1
	*Simplicillium* sp.										1																					1
**Viruses**	*Helicoverpa armigera* NPV												1 ^td^																			1
	*Spodoptera exigua* MNPV													1 ^td^	1 ^td^		1 ^td^															3
	*Spodoptera frugiperda* MNPV							2 ^t^														1 ^d^										3
**Total**		1	10	1	1	7		2	7	2	6	1	4	1	1	1	1	3	5	1	2	1	2	3	1	2	4	1	2	1	10	84

**Table 2:** Effect of entomopathogenic microorganisms on the life cycle of parasitoid wasps; N = significantly negative effect; NE = no significant effect; P = significantly positive effect (*n* = 468 observations extracted from 84 experiments from 43 studies [17,18,19,20,22,23,24,25,26,27,28,29,30,32,34,35,36,37,38,39,41,42,43,44,45,46,47,49,50,51,52,53,54,55,56,57,58,59,60,62,63,64,65]).

Effect of Entomopathogenic Microorganisms on Different Parameters of Parasitoid Wasps.		Parasitism Rate			Emergence Rate			Parasitoid Mortality			Female Sex Ratio			Female Longevity			Male Longevity		
		N	NE	P	N	NE	P	N	NE	P	N	NE	P	N	NE	P	N	NE	P
**Bacteria**	*Bacillus thuringiensis* var. *aizawai*		2			2			2										
	*Bacillus thuringiensis* var. *israelensis*				2			1						1	1			2	
	*Bacillus thuringiensis* var. *kurstaki*		10		2	5			3					2	2	1	2	1	
	*Brevibacillus laterosporus*					2		1						1	1			2	
**Fungi**	*Acremonium sclerotigenum*				1			1											
	*Beauveria bassiana*	53	124	2	77	50		13	97	1	5	48		50	30		45	29	
	*Lecanicillium longisporum*				9	9		36							9			9	
	*Lecanicillium muscarium*	11	7		15	13			6		12	10			1			5	
	*Metarhizium anisopliae*	8	11		13	14		5	5	3		10		11	14		8	16	
	*Metarhizium brunneum*	6	14	1		1			1			1			1			1	
	*Metarhizium robertsii*		1			2			1			2			2			2	
	*Paecilomyces variotii*				1			1											
	*Pandora neoaphidis*																		
	*Simplicillium* sp.				1				1										
**Viruses**	*Helicoverpa armigera* NPV		3	1	1	2								1	2			3	
	*Spodoptera exigua* MNPV	4	2		11	9		2	3										
	*Spodoptera frugiperda* MNPV		4		10	1		10	1			4							
Total		82	178	4	143	110	0	70	120	4	17	75	0	66	63	1	55	70	0

## Data Availability

No new data were created or analysed in this study. Data sharing is not applicable to this article.

## Supplementary Materials

The following supporting information can be downloaded at: https://www.mdpi.com/article/10.3390/pathogens12070957/s1, Figure S1: PRISMA flow diagram of studies looking at combinations of biocontrol agents.
