# Supplementary material for: Entomopathogens and Parasitoids Allied in Biocontrol: A Systematic Review
Source: Pathogens. 2023 Jul 20;12(7):957. doi: 10.3390/pathogens12070957 (PMC10383084; doi:10.3390/pathogens12070957)
Supplement: Supplementary file 1 [file pathogens-12-00957-s001.zip › pathogens-2480220-supplementary.pdf]

Entomopathogens and parasitoids allied in biocontrol: a systematic review

Janique Koller [1,3], Louis Sutter [1], Jérémy Gonthier [2], Jana Collatz [2] and Lindsey Norgrove [3,*]

1 Agroscope, Plant-Production Systems, Route des Eterpys 18, 1964 Conthey, Switzerland
2 Agroscope, Agroecology and Environnment, Reckenholzstrasse 191, 8046 Zurich, Switzerland
3 Bern University of Applied Sciences (BFH), School of Agricultural, Forest and Food Science (HAFL), Länggasse 85, 3052 Zollikofen, Bern, Switzerland
* Correspondence: lindsey.norgrove@bfh.ch

PRISMA 2020 Flow diagram

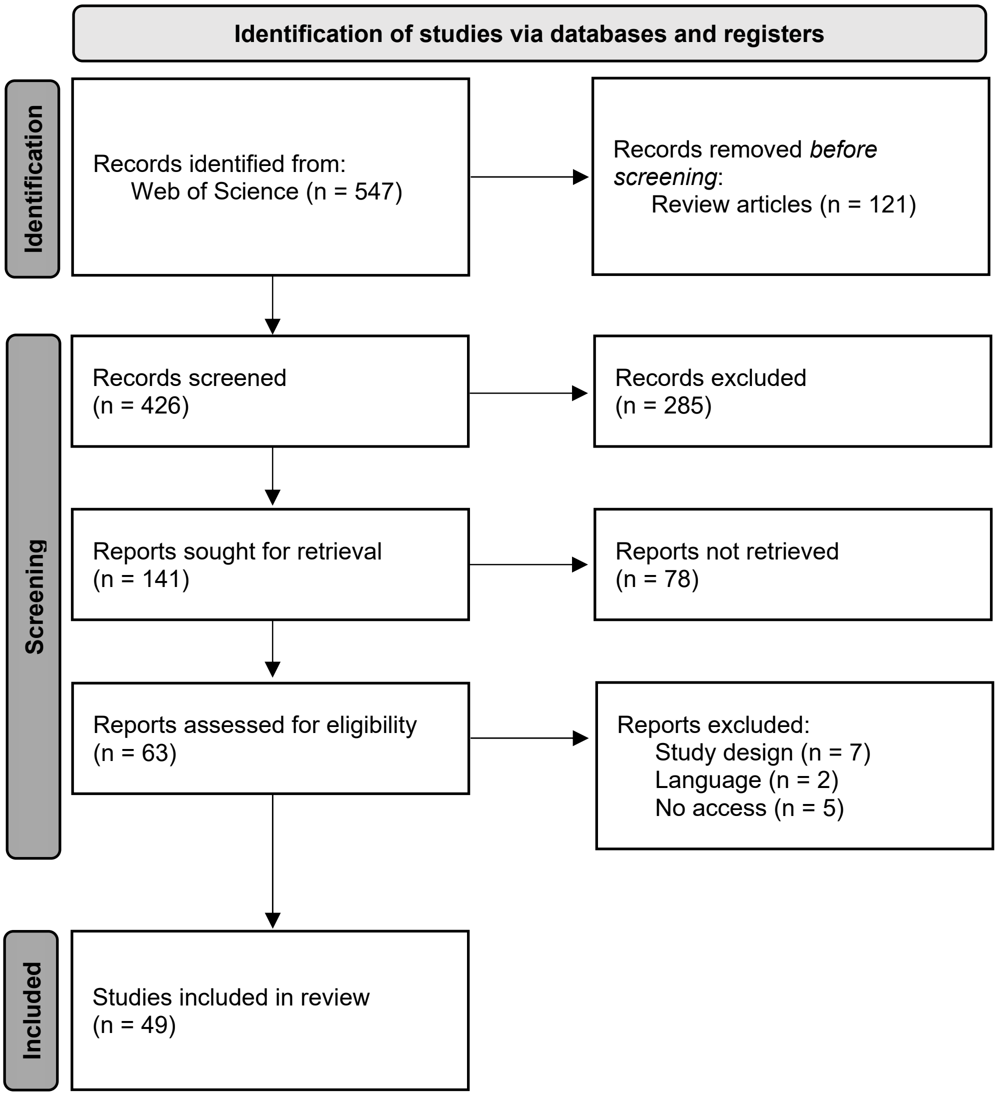

Figure S1 PRISMA flow diagram detailing screening process of articles included in meta-analysis. Procedure adapted from Page et al. 2021.
